# Expression of neuroimmune semaphorins 4A and 4D and their receptors in the lung is enhanced by allergen and vascular endothelial growth factor

**DOI:** 10.1186/1471-2172-12-30

**Published:** 2011-05-19

**Authors:** Elizabeth P Smith, Kathleen Shanks, Michael M Lipsky, Louis J DeTolla, Achsah D Keegan, Svetlana P Chapoval

**Affiliations:** 1 Center for Vascular and Inflammatory Diseases, University of Maryland School of Medicine, 800 West Baltimore St., Baltimore, Maryland 21201, USA; 2 Department of Pathology, University of Maryland School of Medicine, 10 South Pine St., MSTF, Baltimore, Maryland 21201, USA; 3 Department of Microbiology and Immunology, University of Maryland School of Medicine, 685 West Baltimore St., HSF-I, Suite 380, Baltimore, Maryland 21201, USA; 4 Program in Oncology, Marlene and Stewart Greenebaum Cancer Center, University of Maryland School of Medicine, 22 South Greene St., Baltimore, Maryland 21201, USA

## Abstract

**Background:**

Semaphorins were originally identified as molecules regulating **a **functional activity of axons in the nervous system. Sema4A and Sema4D were the first semaphorins found to be expressed on immune cells and were termed "immune semaphorins". It is known that Sema4A and Sema4D bind Tim-2 and CD72 expressed on leukocytes and PlexinD1 and B1 present on non-immune cells. These neuroimmune semaphorins and their receptors have been shown to play critical roles in many physiological and pathological processes including neuronal development, immune response regulation, cancer, autoimmune, cardiovascular, renal, and infectious diseases. However, the expression and regulation of Sema4A, Sema4D, and their receptors in normal and allergic lungs is undefined.

**Results:**

Allergen treatment and lung-specific vascular endothelial growth factor (VEGF) expression induced asthma-like pathologies in the murine lungs. These experimental models of allergic airway inflammation were used for the expression analysis of immune semaphorins and their receptors employing immunohistochemistry and flow cytometry techniques. We found that besides accessory-like cells, Sema4A was also detected on bronchial epithelial and smooth muscle cells, whereas Sema4D expression was high on immune cells such as T and B lymphocytes. Surprisingly, under inflammation various cell types including macrophages, lymphocytes, and granulocytes in the lung expressed Tim-2, a previously defined marker for Th2 cells. CD72 was found on lung immune, inflammatory, and epithelial cells. Bronchial epithelial cells were positive for both plexins, whereas some endothelial cells selectively expressed Plexin D1. Plexin B1 expression was also detected on lung DC. Both allergen and VEGF upregulated the expression of neuroimmune semaphorins and their receptors in the lung tissue. However, the lung tissue Sema4A-Tim2 expression was rather weak, whereas Sema4D-CD72 ligand-receptor pair was vastly upregulated by allergen. Soluble Sema4D protein was present in the lung lysates and a whole Sema4A protein plus its dimer were readily detected in the bronchoalveolar (BAL) fluids under inflammation.

**Conclusions:**

This study clearly shows that neuroimmune semaphorins Sema4A and Sema4D and their receptors might serve as potential markers for the allergic airway inflammatory diseases. Our current findings pave the way for further investigations of the role of immune semaphorins in inflammation and their use as potential therapeutic targets for the inflammatory lung conditions.

## Background

Semaphorins compose a large family of secreted and membrane-bound glycoproteins that are divided into eight subclasses, 1 to 7 and viral [1,2]. They were first identified in the nervous system with a defined function as axon guidance molecules. All semaphorins contain a homologues sequence of approximately 500 aa in the N-terminal extracellular domain [1,2]. The most characterized receptors for semaphorins are plexins and neuropilins [1,2]. Unrelated to plexins and neuropilins, Tim-2 and CD72 receptors expressed on immune cells interact functionally with the members of class 4 family semaphorins [1,2]. Recent findings have demonstrated a new role for semaphorins and their receptors in Th1/Th2 differentiation and inflammation [2-4].

As it was reported previously, in the immune system Sema4A is preferentially expressed on DC and B cells [1,2,5-7]. Sema4A has three known receptors, Tim-2, Plexin B1, and Plexin D1 [5,6]. Plexin D1 was shown to be expressed on endothelial cells [7], whereas Tim-2 (T cell, Ig domain, mucin domain-2) expression was highly restricted to activated T cells, preferentially to Th2 cells [5,6]. A recent study by Yukawa K and associates [8] has demonstrated that Sema4A can also bind and activate Plexin B1. Sema4D (CD100) was found to be constitutively expressed on T cells and its expression was upregulated with T cell activation [1,2,9-11]. Low levels of Sema4D were detected in naïve DC and B cells [2,12]. Sema4D utilizes two known receptors, Plexin B1 and CD72 [9,11]. Plexin B1 mRNA transcripts were found in multiple tissues [11,13]. The main cellular sources of Plexin B1 are epithelial and endothelial cells [1,6,7]. A recently published study showed that epithelial cells in the lung co-express Sema4D and Plexin B1 as determined by immunohistochemistry [13]. Moreover, it was proposed that Sema4D uses Plexin B1 as a receptor only in nonlymphoid tissues [14]. CD72, a 45 kDa type II transmembrane protein belonging to C-type lectin family, has been described as a major lymphocyte receptor for Sema4D [1,2,9-13]. CD72 expression was found to be restricted to B cells, DC and macrophages [1,2,9-12].

Both neuroimmune semaphorins, Sema4A and Sema4D, have complex and critical roles in the immune response that can not be compensated by other family members [1,2,6-13]. Previous studies have demonstrated that Sema4A and Sema4D mRNA transcripts are expressed in many tissues including the lung [5,9-14]. However, the cell types expressing Sema4A and Sema4D ligands in the lung tissue were not clearly defined. To define it, we performed the analysis of lung tissue and cell subset's immune semaphorin and their receptor expression in steady state and allergic inflammatory conditions. For the latter, we used transgenic (tg) mice over-expressing VEGF molecule in a lung-specific manner [15] or WT mice treated with allergen [16]. We show that allergen-treated WT mice display the lung tissue pathology reminiscent to that observed in human asthma with the dominating Th2-driven eosinophilic lung infiltration. We also show here and in our previous report [15] that lung VEGF overexpression induces asthma-like tissue alterations including inflammation, parenchymal and vascular remodeling, edema, mucus metaplasia, and myocyte hyperplasia.

We found the prevalent expression of Sema4D and especially CD72 in the mouse lung which was not restricted to immune cells only but was also found on different subsets of lung resident and inflammatory cells. In addition and importantly, we report here a complimentary cell distribution of these molecules (T cells, B cells, MHCII+ cells co-express a ligand and a receptor) what might be important for the corresponding cell autocrine regulation. The expression of Sema4A and Tim-2 was weak in the lung but was prominent in the lymphoid tissue. However, under acute allergic inflammation Sema4A protein and its dimer were readily detected in the BAL fluids. We found both plexins being expressed on epithelial cells with a prominent upregulation of the epithelial cell Plexin B1 expression under inflammation. Allergen-induced Sema4A expression was detected on the lung underlining epithelium smooth muscle cells suggesting its potential critical role in the smooth muscle function. We also report here that lung DC express Plexin B1 suggesting its role in their activation and/or function. Based on the previously defined specific molecule expression pattern [1-12] and our current results, we conclude that the Sema4A-Tim-2 and Sema4D-CD72 interactions may efficiently occur in both, lung and local lymphoid tissues. We also conclude that the interactions of neuroimmune semaphorins with Plexins might have the specific functional outcomes in the lung as these pairs are co-expressed on several subsets of lung resident and immune cells. As an example, lung epithelial cells co-express Sema4A and both Plexins and lung dendritic cells co-express Sema4D and Plexin B1. As immune semaphorins have been shown to critically non-redundantly regulate the immune response to antigen *in vitro *and *in vivo*, a detailed analysis of their specific tissue expression is an important step in understanding their role in the cell-cell interaction and function and in the target tissue/organ disease regulation. Our data set the ground for further investigations of immune semaphorin modulation and function in lung diseases.

## Methods

### Mice

C57BL/6 mice were obtained from Jackson Laboratory. VEGF tg mice [15] were provided by Jack A. Elias (Yale University). All experiments with the mice were performed in compliance with the principles and procedures outlined in the NIH Guide for Care and Use of Animals and were approved by the University of Maryland School of Medicine Animal Care and Use Committee.

### OVA-induced experimental asthma protocol

C57BL/6 mice (WT mice) were treated with OVA (grade V, Sigma) as described previously [16] and detailed in the Additional files section.

### VEGF-induced lung inflammatory response

VEGF tg mice and WT animals were introduced to the doxycyclin-containing water (0.5 g/L) for 7 days [15].

### Bronchoalveolar lavage cell composition

BAL and lung tissues were obtained from euthanized mice and processed as previously described [17,18]. Cytospin preparations were made with 200 μL of BAL fluid (Cytospin 2; Shandon Inc., Pittsburgh, PA) and stained with Diff-Quick (Dade Behring, Deerfield, IL). The differential cell counts were determined from 4 high-power fields.

### RT-PCR

Conventional (myeloid) DC (cDC) were sorted from lungs of WT and VEGF tg mice as described previously [19]. Cells were then washed in medium at 1200 rpm for 5 min at 4°C. Total RNA (tRNA) was extracted from cell pellet using TRIzol reagent (Invitrogen Life Technologies, Carlsbad, CA) and chloroform isolation procedure combined with the RNAeasy mini kit (Qiagen, Velencia, CA) according to manufacturer's instruction. One μg of tRNA was transcribed into first strand cDNA using SuperScript kit (Invitrogen Life Technologies, Carlsbad, CA). Then 500 ng of cDNA was used for PCR amplification. PCR reaction products were run on 1.5% agarose gels and visualized using ethidium bromide. Quantification of the target gene expression was done by comparison with the expression of β-actin. The gene specific primers for RT-PCR were designed using the Primer3 Input 0.4.0 software. The amplicon specificity was verified by gel running and/or by sequencing.

### Histochemistry and immunohistochemistry

Frozen sections from WT and VEGF tg mouse lungs were prepared as described in Additional files 1 and 2. Paraffin-embedded tissues from PBS- or OVA-treated mouse lungs (n = 3-4 mice per experimental group in 3-4 independent experiments) were prepared as described in the Additional files. Unlabeled Abs against intracellular portions of Sema4A (clone C-20) and Sema4D (clone C-19), extracellular portions of Plexin B1 (N-18) and D1 (E-13) were obtained from Santa Cruz. Unlabeled anti-mouse CD72 (AF-1279) and Tim-2 (AF-1885) Abs were obtained from R&D Systems. Stainings were visualized as detailed in the Additional files. Staining specificity was assessed by evaluation of the appropriate isotype control Ab stain. According to the Ab's datasheets, Abs were generated by immunization of corresponding hosts with a corresponding purified recombinant protein or a protein fragment. Each Ab was then purified using the relevant Ag chromatography. The specificity and quality of Abs used in our assays was assessed by the manufacturer in Western blot using lysates of Ag-transfected either EL-4 or 293T cells and in direct ELISA.

### Flow cytometry

Lung tissue collagenase (Worthington Biochemical Corp., Lakewood, NJ) -DNAse (Roche, Mannheim, Germany) digests were performed (n = 2-3 mice per experimental group in 3-4 independent flow cytometry experiments) and stained for FACS analysis as described previously [19]. The following mAbs obtained from BD Biosciences Pharmingen (San Diego, CA) were used: anti-I-Aβ^b^-PE (AF6-120.1), anti-CD3-FITC or -PE (145-2C11), anti-CD4-FITC or -PE (GK1.5), anti-CD8α-PerCP (53-6.7), anti-CD11c-APC or -FITC (HL3), anti-B220/CD45R-FITC (RA3-6B2), anti-GR1-PE (Ly-6G and Ly-6C), anti-NK1.1-APC (PK136). Anti-Mac-1-PE-Cy-5 (CD11b/CD18) Ab was obtained from Cedarlane Laboratories and anti-Plexin B1 Ab (A-8) was purchased from Santa Cruz. For the detection of CD72 and Tim-2, we employed Abs used for IHC and, in addition, FITC-labeled anti-CD72.1 (ab25029, Abcam) and biotinylated anti-mouse Tim-2 Ab (RMT2-1, Biolegend). Biotin-labeled anti-Sema4A (KL-1) and anti-Sema4D (BMA-12) were obtained from eBioscience. FITC-labeled Sema4A (5E3) and Sema4D (BMA-12) Abs were obtained from Medical & Biological Laboratories, Japan. SAV-PerCP and SAV-FITC were used as the second step reagents for biotinylated Abs. FITC-conjugated or biotinylated rat IgG1 (R3-34 or A110-1), mouse IgG1 (Sigma), rat IgG2a (R35-95), rat IgG2b (R35-38) were used as isotype controls. Plasmacytoid DC visualization was performed as described in. Where necessary, cells were preincubated with anti-CD16/CD32 (2.4G2) mAb for blocking cell surface FcR. Cells were analyzed on FACSCalibur (Becton Dickinson, San Jose, CA) flow cytometer using either CELLQuest or FlowJo software.

### Intracellular staining

Single cell suspensions from the lungs of PBS- or OVA-treated mice were washed and placed in FACS buffer with anti-CD16/CD32 Ab (BD Biosciences) to block nonspecific binding (1 μg Ab/1 mln cells). Cell surface molecule staining was performed using anti-CD11c-APC, anti-I-Aβ^b^-PE, and anti-Mac-1-PE-Cy-5 Ab specified above. Cell were washed, permeabilized, and stained with FITC-anti-Sema4A (5E3) as described in the Additional files.

### Western blot

Whole lung lysate preparation, protein extraction, basic Western blot procedure, and protein detection were previously described [20]. Anti-Sema4D primary Ab (clone Y-20) and HRP-conjugated donkey anti-goat secondary Ab employed in this study were purchased from Santa Cruz Biotechnology. 15 microliters of BAL fluids obtained from either PBS- or OVA-challenged WT mice were used for a detection of Sema4A expression using anti-Sema4A primary Ab from MBL (clone 5E3) and HRP-labeled donkey anti-mouse secondary Ab (sc-2414, Santa Cruz). Both blots were performed in reducing conditions using either the 4-20% Tris-HCl Gel from BioRad (for Sema4D) or 10% Precise Protein Gel from ThermoScientific (for Sema4A) with the Precision Plus molecular weight marker (BioRad). ECL detection reagents kit (Amersham Biosciences) was used according to the manufacturer's instruction.

### Statistics

Data were summarized as mean ± SEM. To calculate significance levels between experimental groups, Student's t test (Microsoft Excel) was performed.

## Results

Monitoring of specific molecule expression was done in C57BL/6 (WT) mice treated with either PBS or allergen and in VEGF tg mice with 7 days of transgene induction as described in Methods.

### Allergen and VEGF induce asthma-like inflammatory responses

**Figure 1:**
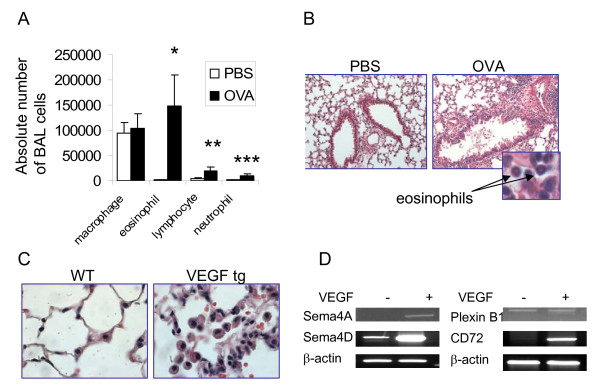
**Lung exposure to either allergen or VEGF induces asthma-like inflammatory responses**. (A-B) WT mice were exposed to OVA as described in the Methods section. Significant influx of eosinophils, lymphocytes, and neutrophils into BAL of allergen-treated WT mice was found compared to PBS-treated counterparts (*p < 0.025, 0.045, and 0.03, correspondingly) (A). Lung tissue inflammatory response observed on histology slides corresponds that in BAL (magnification x20, for insert 100x) (B). (C-D) Lung VEGF expression induces local inflammation and upregulation of selected neuroimmune semaphorin and receptor molecules in sorted lung conventional DC. H&E staining of lungs from WT and VEGF tg mice given DOX water for 7 days (magnification 40x) (C). Sorted mouse lung cDC were subjected to tRNA extraction followed by cDNA preparation and PCR with gene-specific primers. Note **a **strong upregulation of Sema4D and CD72 in lung cDC by VEGF (D).

Allergen treatment of WT mice induced a significant influx of inflammatory cells into the lungs measured by cell counts in the BAL and the H&E staining of lung tissue (Figure 1 A and 1B). Eosinophils were found to dominate the cellular infiltrate in the BAL of OVA-treated mice mounting to 147,233 ± 61,999 cells whereas they were absent in the BAL of PBS-treated mice (0 ± 0). Lung tissue histochemistry pictures corresponded that of BAL cell composition (Figure 1 B).

We reported previously that VEGF tg mice developed asthma-like phenotype with inflammation, parenchymal and vascular remodeling, edema, mucus metaplasia, myocyte hyperplasia and airway hyperresponsiveness [15]. The tissue changes begin to occur in tg mice as quickly as being 2 days on DOX-containing water and progress significantly with time [15]. We show here a significant lung pathology observed in tg mice on day 7 after tg expression initiation (Figure 1 C) with multiple large inflammatory macrophages noted in the alveolar spaces.

We also showed previously how lung VEGF expression functionally affects local conventional DC for the transition from the innate response to a Th-2-type inflammatory response [19]. VEGF regulates many innate immunity molecules in cDC. To study if VEGF has an effect on lung cDC expression of immune semaphorins and their receptors, we analyzed mRNA levels for selected specific semaphorins and receptors by RT-PCR (Figure 1 D). cDC from VEGF tg mice exhibit increased mRNA expression of Sema4A, Sema4D, and CD72 as compared to analogous cells sorted from WT mouse lungs. Upregulation of Sema4D was especially prominent, whereas the expression of Plexin B1 was not changed, and Sema4A was slightly induced in tg cDC. Therefore, VEGF overexpression in the lung induces an upregulation of specific neuroimmune semaphorins and their receptors. Upregulation of neuroimmune semaphorin expression is a characteristic of activated DC [1,2,5,10,12]. This observation let us to hypothesize that VEGF- and allergen-induced lung tissue alterations are, at least in part, immune semaphorin-dependent. Thus, we focused our study on the expression of immune semaphorins and their receptors in the lung tissue and cells.

### Regulation of lung Sema4A and Sema4D expression by allergen and VEGF

**Figure 2:**
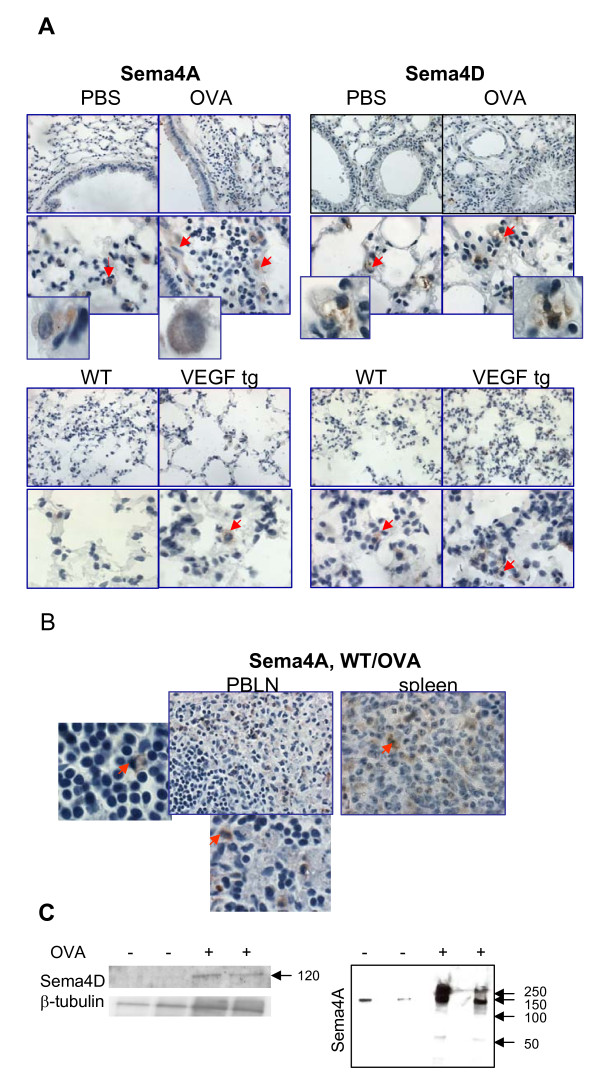
**Lung tissue Sema4A and Sema4D expression and their regulation by allergen and VEGF**. (A) Formalin-fixed paraffin-embedded lung tissue sections (top microphotographs) were deparaffinized and immunohistochemistry was performed employing Abs for the intracellular portions of Sema4A and Sema4D molecules. Bottom photomicrographs show immunohistochemistry on frozen lung tissue sections obtained from VEGF tg mice and control WT mice being on DOX water for 7 days. Sema4A expression in PBS-treated lungs was mainly limited to macrophages and dendritic like-cells. Tissue Sema4A expression was upregulated with allergen treatment and VEGF exposure and detected on APC-like cells and smooth muscle cells (both cell types marked with red arrows). Inserts show high magnification fields (100x) with marker-positive cells. Sema4D expression was detected in cell bundle-like shapes (red arrows, inserts) in the lungs obtained from both PBS- and OVA-treated mice. OVA treatment did not significantly modulate tissue levels of Sema4D. (B) Sema4A was abundantly expressed on APC-like cells (inserts, red arrows) in lymphoid tissue of allergen-treated WT mice. (C) Soluble Sema4D protein (120kDa) was detected in lung tissue lysates obtained from OVA-treated mice. Whole Sema4A protein (150 kDa), dimer (>250 kDa), and weak soluble Sema4A (120 kDa) were detected in BAL fluids of OVA-challenged mice. Arrows point to the molecular weight protein standard reference bars.

Lung tissue immunohistochemistry with Ab for the intracellular portion of Sema4A has shown that significant number**s **of cells with macrophage- and DC-like morphology were positive for Sema4A in the control PBS-treated mouse lungs (Figure 2 A). Sema4A expression in the lung was increased during allergen-induced inflammation due to the influx of the marker-positive cells (3.2 ± 0.9 vs 6.2 ± 1.4 cells/high power 100x field, p < 0.042, PBS- vs OVA-treated mice). Bronchial epithelial cells showed a weak Sema4A expression whereas underlining epithelium smooth muscle cells were strongly Sema4A-positive (Figure 2 A). Isotype control Ab did not stain the tested tissues (Additional files, Figure 1) indicating Ab specificity and a definite upregulation of Sema4A in the allergen-treated lungs. In contrast to its relatively low expression in the lung tissue, Sema4A was readily detected in the lymphoid tissue of allergen-treated WT mice (Figure 2 B) and its expression was restricted, as expected, to APC-like cells.

Using Ab for C-terminus of Sema4D for IHC, we found an unusual pattern of Sema4D staining which was not targeted to the cell cytoplasm but rather to cell bundle-like shapes (Figure 2 A) in the lungs obtained from both PBS- and OVA-treated mice. Its tissue expression was not much modulated by allergen treatment as assessed by IHC with this Ab (3.0 ± 0 vs 4 ± 0.6 cells/high power 100x field, p < 0.08, PBS- vs OVA-treated mice).

Similarly to C57BL/6 mice, in the VEGF tg mouse lungs a substantial number of accessory-like cells was stained positively for Sema4A (Figure 2 A). Tg expression upregulated the tissue expression of this semaphorin as there were more Sema4A+ cells in tg lungs. Sema4D lung tissue expression was mainly detected in APC-like cells and was significantly upregulated by VEGF (Figure 2 A). This upregulation included both an increase in the marker-positive cell number**s **(3.2 ± 1.8 vs 8.4 ± 3.9 cells/high power 100x field, p < 0.04, WT vs VEGF tg mice) and a brighter marker expression per cell in tg lungs as defined by the intensity of staining.

It has been reported that under inflammation **a **membrane-bound Sema4D gets cleaved by MT1-MMP and sheds from the cell surface [19]. Indeed, employing Ab to **a **soluble form of Sema4D (Y-20, Santa Cruz), we found this molecule to be present in the lung tissue lysates of OVA-treated but not PBS-treated WT mice (Figure 2 C). Similarly, the soluble 120 kDa Sema4A form was detected in the culture supernatants of Sema4A-transfected cells (7). Therefore, it has been proposed that Sema4A also sheds from the cell surface under inflammatory conditions. When we used the BAL fluids from either PBS- or OVA-challenged mice in Western blot under conditions defined in the Methods section, we found increased levels of the a whole 150 kDa Sema4A protein in the OVA-treated mice as compared to PBS-treated counterparts (Figure 2 C). A weak 120 kDa band corresponding soluble Sema4A protein was also detected under inflammation, however, a band corresponding Sema4A dimer (>250 kDa) was much more prominent.

**Figure 3:**
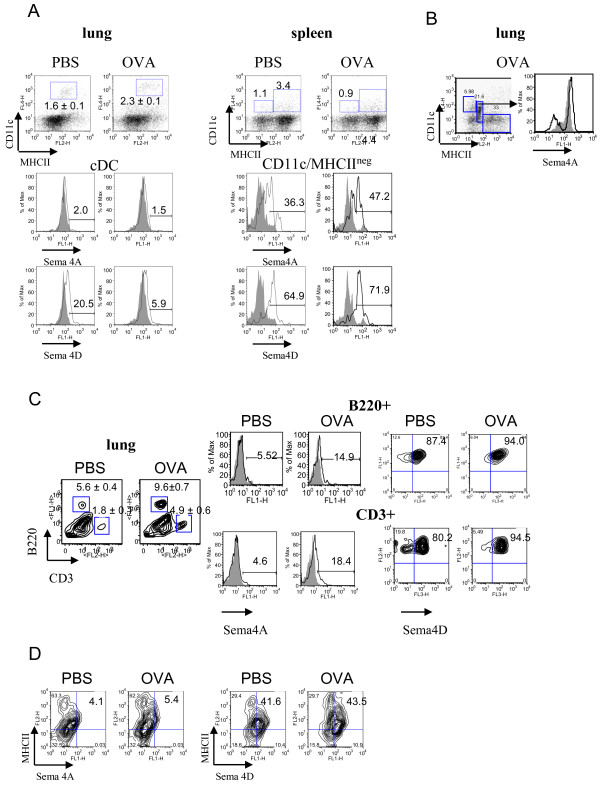
**Regulation of Sema4A and Sema4D expression on lung immune cells by allergen and VEGF**. Flow cytometric detection of Sema4A and Sema4D expression on lung and spleen DC (A-B), lung T and B cells (C), and MHCII+ cells (D). Single cell suspensions were prepared as described in Methods. Conventional DC were identified by staining cells with anti-CD11c, -MHCII, and -CD11b Abs used for lung cDC detection. Highly fluorescent macrophages were gated out from the further analysis as large cells on FSC-SSC. (A) No cell surface Sema4A and low Sema4D expression was found on lung cDC. Spleen CD11c+MHCII^neg ^DC subset demonstrated high Sema4A and Sema4D expression which was further upregulated by allergen. (B) Intracellular Sema4A was targeted to a specific population of CD11c^intermed^/MHCII^low ^cells under inflammatory conditions. (C-D) Low Sema4A and high Sema4D expression was detected on lung B220+ cells, CD3+ cells, and MHCII+ cells.

Flow cytometry analysis of lung single cell suspensions has shown that, in contrast to lymphoid organs, there was no significant Sema4A expression on lung APC surface (Figure 3 A). This included lung cDC which were defined as CD11c+MHCII^low ^cells in PBS-treated mice and as CD11c+/MHCII+ cells in OVA-treated mice [19]. Low number of lung plasmacytoid (p)DC, which were defined as CD11c^intermed^B220+ cells [19], were found to express Sema4A (from 7.5% to 17.9%, PBS vs OVA-treated mice, data not shown). Intracellular staining of the lung cells obtained by an enzymatic tissue digest has shown that Sema4A expression was predominantly targeted to a specific cell subset, namely CD11c^intermed ^MHCII^low ^cells (Figure 3 B) which could potentially include pDC, precursors of conventional lung DC, and a subset of lung macrophages [19,21,22]. Of note, neither CD11c+MHCII- cells nor CD11c-MHCII+ cells evaluated showed measurable levels of intracellular Sema4A (data not shown). Initial low level of lung cDC Sema4D expression was further downregulated after OVA treatment (Figure 3 A) possibly due to the previously described molecule shedding from the cell surface in inflammation [11,23]. Indeed, the presence of an extracellular portion of Sema4D (120 kB) in allergic but not control lungs was confirmed by Western blot employing tissue lysates (Figure 2 C).

As a control for the flow cytometry study of neuroimmune semaphorin expression on lung cells we used spleen MNC where their expression was partially defined previously [1,2,5,7-12]. We identified two spleen DC subpopulations based on a dual marker for DC, CD11c and MHCII, namely CD11c+MHCII- and CD11c+MHCII+ (Figure 3 A). Interestingly, we have found that in contrast to lung DC, both spleen CD11c+ populations express a substantial level of Sema4D and Sema4A (Figure 3 A and data not shown). CD11c+MHCII+ cells did not show any modulation of immune semaphorin expression by allergen (data not shown). CD11c+MHCII- cells, which might include DC precursors, immature DC, and/or pDC [24,25] had significantly higher constitutive levels of both molecule**s **when compared to CD11c+MHCII+ cells. Allergen exposure increases the corresponding marker expression on these DC (from 36.3% to 47.2% and from 64.9% to 71.9%, PBS vs OVA, Sema4A and 4D, respectively, Figure 3 A).

As expected, lung T cells (CD3+ cells) did not express much of Sema4A in both steady-state and inflammatory conditions (Figure 3 C). In contrast, Sema4D molecule was readily detected on T cells (Figure 3 C). Surprisingly, lung B cells (B220+ cells) in PBS-treated mice were also found to be highly Sema4D+ (Figure 3 C), although it has been reported previously that Sema4D is weakly expressed on naïve B cells [2,12]. In PBS-treated mice, around 45% of MHCII+ cells in the lung co-expressed Sema4D whereas Sema4A expression on these cells was negligent (Figure 3 D). The levels of cell expression for both molecules did not change significantly with OVA treatment. NK1.1+ cell number increased in the inflamed lung (5.0% and 10.3% of total cells, PBS- vs OVA-treated mice). These cells showed low Sema4A (around 11%) but high Sema4D expression (63.8%) which was downregulated by OVA (data not shown).

Therefore, the overall presence of a membrane-bound Sema4A molecule in the lung tissue is low. Thus, its contribution to the lung inflammatory response regulation is most probably restricted to the local lymphoid tissue where it is abundantly expressed on CD11c+ cells. In contrast, Sema4D is highly expressed on lung immune cells (T and B cells), and, to a lower extend, on lung MHCII+ cells which, in addition to the lung professional APC, might include epithelial cells, fibroblasts, mast cells, macrophages, and, under inflammation, granulocytes [26-29]. Thus, Sema4D can potentially regulate lung inflammatory response both locally and in the lymphoid tissue.

### Regulation of lung Tim-2 and CD72 expression by allergen and VEGF

We next performed IHC and flow cytometry studies to define the lung expression of Tim-2 and CD72, corresponding Sema4A and Sema4D immune cell receptors.

**Figure 4:**
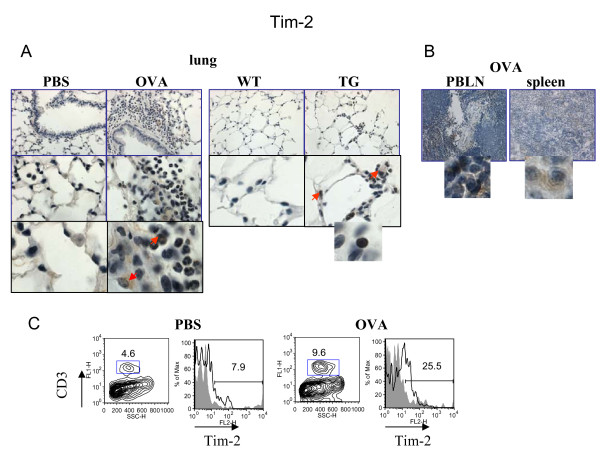
**Regulation of lung Tim-2 expression by allergen and VEGF**. Immunohistochemical (A-B) and flow cytometric (C) detection of Tim-2 expression in lung (A) and lymphoid (B) tissues, and on lung T cells (C). (A-B) Note positive Tim-2 staining on different lung cells besides lymphocytes in allergen- or VEGF-exposed mouse lungs. Red arrows point to Tim-2+ APC-like cells and granulocyte. Inserts show high magnification fields (100x) with marker-positive cells (lymphocytes). (C) Flow cytometry analysis of cells from lung enzymatic digests obtained from PBS- and allergen-treated mice showed an increase in lung Tim-2+ T cells in OVA-treated mice. The histograms show the percentage of Tim-2+CD3+ cells in the lung (clear histogram) as compared to the appropriate isotype control stained CD3+ cells (gray histogram).

As it has been shown previously, Tim-2 is a marker for activated T cells, preferentially Th2 cells [5-7], whereas CD72 is found to be expressed on B cells and DC [9-12]. To our surprise, using Ab for IHC specified in the Methods section, Tim-2 staining was detected on many cells in the lungs of allergen-treated mice (Figure 4 A) including inflammatory macrophages and a subset of granulocytes. This staining was specific when compared to isotype control Ab staining (Additional files, Figure 1). Some lymphocytes were also Tim-2-positive. In VEGF tg mice Tim-2 was targeted predominantly to the tissue lymphocytes with a weaker staining detected in macrophages and granulocytes (Figure 4 A). In contrast to the lung tissue, strong Tim-2 expression was readily detected in spleen and local lymph nodes of allergen-treated WT mice (Figure 4 B). Flow cytometry data had shown an increase in Tim-2-positive T cells in allergic lung (mean of fluorescence intensity from 7.94 to 25.5%, PBS- vs OVA-treated mice, respectively) (Figure 4 C). The physiological mean of Tim-2 expression on other cell types besides T cells has to be determined.

**Figure 5:**
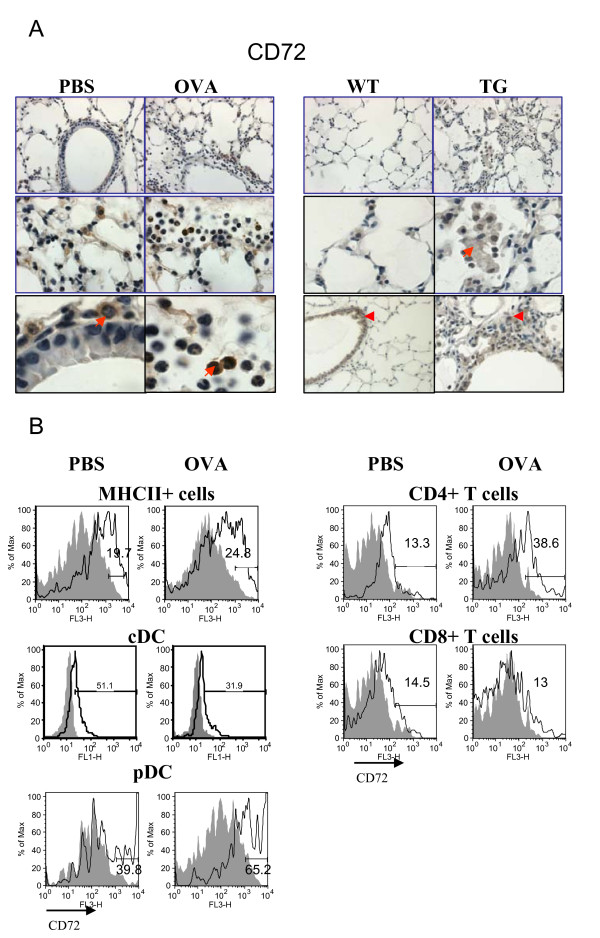
**Regulation of lung CD72 expression by allergen and VEGF**. Sema4D receptor CD72 expression in mouse lung tissue and cells was analyzed by immunohistochemistry and flow cytometry (A-B). (A) Formalin-fixed paraffin-embedded lung tissue sections obtained from either control or OVA-treated WT mice were deparaffinized and immunohistochemistry was performed using appropriate Abs as described in Materials and Methods. Frozen lung tissue sections were used for VEGF tg mice. CD72 is the most abundantly expressed molecule in the lungs of PBS-treated mice. Its expression is further upregulated with OVA treatment. Note that subsets of inflammatory granulocytes and lymphocytes express CD72 (red arrow). In VEGF tg lungs CD72 expression was mainly targeted to inflammatory macrophages (red arrow). Red arrowhead points to the lung epithelial cell CD72 expression. (B) CD72 is expressed on lung MHCII+ cells including DC and is upregulated by OVA. Both CD4+ and CD8+ T cells in the lung express CD72 at low level which is further upregulated by allergen on CD4+ T cells.

IHC demonstrated an abundant CD72 expression in control murine lungs which was greatly pronounced with inflammation (Figure 5 A). Lung accessory-like cells (red arrow on the tissue picture obtained from a PBS-treated mouse) and alveolar type II-like cells (middle photomicrograph) were CD72-positive. In addition, we found CD72 to be expressed on bronchial epithelial cells in large bronchi but rarely in smaller bronchi. Alveolar macrophages were also CD72-positive. Under OVA-induced inflammatory conditions, some additional cells such as subsets of lymphocytes, granulocytes, and inflammatory macrophages were also found to express CD72 (Figure 5 A). A pattern of CD72 expression in WT control mice being on DOX water was similar to that observed in PBS-treated WT mice although the staining appears weaker (Figure 5 A). In VEGF tg mice the expression of CD72 was noted mainly on lung inflammatory macrophages and epithelial cells in large bronchi (Figure 5 A). Flow cytometry assessment of MHCII+ cells, DC and T cells showed the level of CD72 cell expression and its upregulation by allergen (Figure 5 B). As it has been reported previously, in the lymphoid tissue CD72 is expressed on B cells, DC, and a small fraction of activated T cells [1,2]. Indeed, in the murine lungs MHCII+ cells express Sema4D receptor CD72 (Figure 5 B). In accord with our IHC data, CD72 was also found to be expressed on lung DC and it was positively regulated by allergen. Interestingly, only a fraction of lung T cells express CD72 which further increase**s **for CD4 + but **not **for CD8+ T cells after allergen treatment. Therefore, CD72 can potentially be a useful marker for a specific cell subset's activation status.

### Regulation of lung Plexin D1 and B1 expression by allergen and VEGF

**Figure 6:**
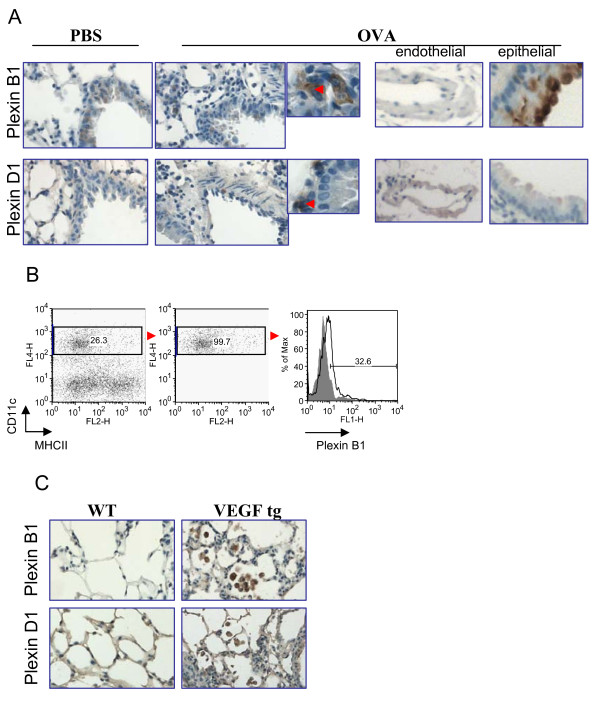
**Plexin B1 and Plexin D1 expression in the mouse lung tissue and its regulation by either allergen or transgenic VEGF exposure**. (A) Plexin B1 expression was detected on selected bronchial epithelial cells in control WT mice with increased expression in allergen-treated mice. Under inflammatory condition, its expression was detected on a number of APC-like cells (insert, red arrow). Plexin D1 expression was restricted to endothelial and a number of bronchial epithelial cells. In addition, Plexin D1 was found on underlining epithelium cells (insert, red arrow). (B) Plexin B1 expression on lung conventional DC under inflammation. WT mice were treated with OVA as described in Materials and Methods and lung cDC were identified as CD11c+MHCII+ cells which were gated for further analysis. The histograms show the percentage of Plexin B1+ gated cDC (clear histogram) as compared to the appropriate isotype control stained cells (gray histogram). (C) Notable upregulation of the expression of both Plexins in VEGF tg lungs was mainly targeted to inflammatory macrophages found in the interstitial spaces.

Lastly, we performed IHC to define the lung tissue distribution of Plexins D1 and B1, corresponding Sema4A and Sema4D non-immune cell receptors. Previous studies have shown both plexins to be expressed on endothelial cells [26,27]. However, we showed here that mouse lung endothelial cells did not stain positively for Plexin B1 (Figure 6 A). Around 20% of the lung bronchi and bronchioles were found to express Plexin B1 while most of them were marker-negative. OVA treatment upregulated this level to more than 50% of corresponding **airways **(Figure 6 A). In addition, Plexin B1 expression was noted on APC-like cells in the lung tissue. This was supported by **the **flow cytometry study which demonstrated Plexin B1 expression on lung cDC (Figure 6 B).

In allergen-treated WT lungs, Plexin D1 expression was mostly targeted to endothelial cells (Figure 6 A). Some large bronchi demonstrated a weakly-positive Plexin D1 staining in the apical portion of lining epithelium if compared to Plexin B1 expression. In addition, some APC-like cells in the bronchi basement membrane were found to be highly positive for this Sema4A receptor under inflammatory conditions. VEGF-induced lung inflammatory response clearly correlated with more local Plexin expression as both Plexins were upregulated in VEGF tg lungs (Figure 6 C). Whereas Plexins were found predominantly on the epithelium of large bronchi, notable inflammatory macrophage expression of both molecules was found in tg mice.

## Discussion

In this manuscript we demonstrated, for the first time, the expression of neuroimmune semaphorins and their receptors in the lung tissue and subsets of cells and compared it to the previously published and obtained in our laboratory results on the expression of these molecules in the immune tissues. We show here that the lung tissue expression of neuroimmune semaphorins is more complex than that shown previously for the immune and tumor cells [5-14,30,31]. First, the expression of immune semaphorins and their so-called "immune cell receptors" in the lung is not restricted to immune cells but can be also found on many resident and inflammatory cells. For example, the most abundantly distributed in the lung CD72 molecule was found to be present on the subsets of macrophages, granulocytes, and lymphocytes as well as on alveolar macrophages, alveolar type II-like epithelial cells, and bronchial epithelial cells. These IHC results have to be further confirmed using the specific cell surface markers for the named above lung resident cell subsets. Second, we showed a somewhat different distribution of neuroimmune semaphorins and their receptors in the lung tissue as compared to the lymphoid organs. Specifically, we defined that Plexin B1, a common receptor for both Sema4A and Sema4D which was previously reported for epithelial and endothelial cells [1,6,7], is also expressed on lung DC (Figure 6 B). In addition, we detected Th2 cell marker Tim-2 being expressed on several resident and inflammatory cell subsets in the inflamed lung (Figure 4). Finally, we identified the cells in the lung co-expressing a specific ligand-receptor pair suggesting a possible critical role of these molecules in the corresponding cell activation and/or function.

Constitutively high Sema4A expression on the surface of all mouse DC subsets, including BMDC, CD8+DC and CD8-DC was reported previously [32]. We show here that, in contrast to lymphoid organs, Sema4A was practically absent on all lung MHCII+ cells (Figure 3 A and 3D). Its expression was not detected on lung cDC, was very low on lung pDC, T cells and B cells. As on the IHC slides Sema4A expression was found to be mainly targeted to APC-like cells with some cells showing clear dendritic cell morphology (Figure 2 A plus inserts), we performed a flow cytometry assay employing Abs to the cell surface markers for lung cDC, namely CD11c, MHCII, and CD11b [19] and Ab for intracytoplasmic portion of Sema4A for intracellular staining. Using these markers, we found the presence of an intracellular Sema4A in a specific subset of CD11c^intermed^/MHCII^low ^cells under allergic inflammatory conditions. Lung tissue Tim-2 expression was also low (Figure 4 A and 4C). These findings let us conclude that most probably lung tissue Sema4A-Tim-2 interaction, which is critical for T cell activation [32], might not be efficient under the conditions studied. However, Sema4A protein and its dimer were readily detected in the BAL fluids obtained from OVA-challenged WT mice suggesting that it can exert its activity as a locally released molecule. In this study we used a protocol for an acute allergic inflammatory response generation in mice. It is possible that in chronic inflammatory conditions more abundant cell surface Sema4A expression can be detected. Additionally, Sema4A has been reported to be present on endothelial cells [7], however, we could not find it on lung endothelial cells using IHC with **a **corresponding Ab defined in the Methods section. Although lung Sema4A expression was upregulated by either allergen or VEGF, it was still mainly targeted to APC-like cells and underlining bronchial epithelium smooth muscle cells (Figure 2 A). In contrast to its weak expression in the lung tissue, a membrane-bound Sema4A was readily detected in the lymphoid tissues by IHC (Figure 2 B) and specifically on spleen DC by flow cytometry analysis (Figure 3 A and data not shown) where its expression was upregulated in response to OVA treatment on lung CD11c+MHCII- cell subset. Tim-2 was also readily detected in lymphoid organs under allergic inflammatory conditions (Figure 4 B). Therefore, the lymphoid tissue membrane-bound Sema4A-Tim-2 interaction and the lung locally released Sema4A protein and dimer could be critical for the allergic immune response generation and regulation. Surprisingly, we found various cell types expressing low levels of Sema4A receptor Tim-2 in the allergen-treated WT lungs and VEGF-exposed lungs by IHC. The flow cytometry study has shown that more than 25% of lung T cells expressed Tim-2 in OVA-treated mice what corresponded to a 3-fold increase over the baseline expression level found on PBS-treated mouse lung T cells. As Tim-2 is a reported marker mainly for Th2 cells [5,6], our data suggest either that under **the **inflammatory conditions studied Th2 cells represent a minor portion of lung infiltrating T cells or that not all lung infiltrating Th2 cells are Tim-2-positive.

In contrast to the relatively low lung tissue/cell Sema4A and Tim-2 expression, the other neuroimmune semaphorin and its immune cell receptor, namely Sema4D and CD72, were found to be abundantly expressed on many lung cells. A detection of Sema4D by IHC using Ab to its C-terminus showed an unusual cell bundle pattern of molecule distribution (Figure 2 A) whereas flow cytometry study with Ab to its extracellular portion was more definitive in targeting its expression to the specific cell subset**s **(Figure 3 A, C, and 3D; Figure 5 B). Sema4D expression on lung cDC was very low in PBS-treated lungs and was further downregulated by allergen (Figure 3 A). It is currently well established that lung cDC are critical APC for the allergic lung immune response initiation and progression [33,34]. However, it is also well known that Sema4D exists in a soluble form under inflammatory conditions. Therefore, the identified subtle changes in cell-surface Sema4D expression under allergic inflammation might not represent the local actual level of this molecule. Indeed, we found the extracellular portion of Sema4D (120 kDa) in the lung lysates under OVA-induced inflammation but not in steady-state condition (Figure 2 C). High cell surface Sema4D expression was detected on lung MHCII+ cells, T and B cells. MHCII+ cells (including DC) were also found to express Sema4D immune cell receptor CD72 (Figure 5 B). It has been shown previously for B cells and DC that the addition of a soluble Sema4D protein or Sema4D-expressing cells to their cultures significantly enhanced their CD40-induced proliferation and differentiation [10,35]. Therefore, in addition to a direct APC activation through T cell's Sema4D and APC's CD72 [1,2,9-12] following a primary activation signal (either the Ag stimulation or CD40-CD40L mediated signal), there could be a potential autocrine Sema4D-CD72-dependent mechanism of lung APC activation. The release of a soluble Sema4D in the lung microenvironment under inflammatory condition**s **where its receptor CD72 is abundantly expressed on many lung resident and inflammatory cells (Figure 5 A and 5B) could provide an additional important local activation signal for these cells.

Our data demonstrate the specific regulation of lung immune semaphorins and their receptors in the allergen-dependent (OVA-induced) and -independent (VEGF-regulated) allergic airway inflammation. In conclusion, we show here that the expression of immune semaphorins and their receptors in the lymphoid tissue and lung tissue has some similarities as well as some differences which need to be taken in account when designing a strategy for the specific organ/tissue disease intervention based on immune semaphorins as therapeutic molecular targets.

## Conclusions

The understanding of the biology, distribution, and function of neuroimmune semaphorins Sema4A and Sema4D is still in the early stage. Additional studies are needed to define a detailed picture of each molecule and each corresponding receptor regulation of the immune and non-immune tissues and their roles in specific diseases. Nevertheless, a current state of knowledge emerge neuroimmune semaphorins as critical regulatory molecules with multiple distinct functions including immune cell costimulation, regulation of cell morphology, adhesion, and migration. Our study defines Sema4A, Sema4D, and their receptors as potential markers and potential drug targets for allergic airway inflammatory diseases.

## Authors' contributions

EPS performed IHC, KS genotyped the tg mice and dissected control lungs, MML and LJD analyzed lung tissue alterations and IHC, ADK provided financial and mentorial support for the project, SPC designed the study, performed flow cytometry experiments, captured IHC pictures, analyzed the data, and drafted the manuscript. All authors read and approved the final manuscript.

## Supplementary Material

Additional file 1 **Details for the Materials and Methods Section**. This section gives additional details on the following methods used: OVA-induced experimental asthma protocol, histochemistry and immunohistochemistry, and plasmacytoid dendritic cell visualization Click here for file

Additional file 2 **Additional figure and figure legend**. This section shows the photomicrographs of **i**sotype control goat IgG stains of mouse lung tissues. Click here for file
