# Supplementary material for: Expression of neuroimmune semaphorins 4A and 4D and their receptors in the lung is enhanced by allergen and vascular endothelial growth factor
Source: BMC Immunol. 2011 May 19;12:30. doi: 10.1186/1471-2172-12-30 (PMC3118960; doi:10.1186/1471-2172-12-30)
Supplement: Additional file 1 — Details for the Materials and Methods Section. This section gives additional details on the following methods used: OVA-induced experimental asthma protocol, histochemistry and immunohistochemistry, and plasmacytoid dendritic cell visualization [file 1471-2172-12-30-S1.DOC]

**Additional files**

**Additional file 1**

**OVA-induced Experimental Asthma Protocol**

Six- to eight-week-old mice received prime and boost injections of 100 μg/200 μl/mouse OVA in 2 mg Alum (Sigma) in PBS on days 0 and 5. Then, on day 12 mice received 1% OVA/PBS challenge by 40 min nebulization with Invacare Envoy nebulizer (Sanford, FL). All assessments were performed on day 14.

**Histochemistry and Immunohistochemistry**

Lungs from PBS- or OVA-treated mice were inflated with 0.2 ml of 10% formalin, fixed in formalin solution before embedding them into paraffin. For obtaining frozen lung tissue sections, lungs from WT and VEGF tg mice were inflated with 0.2 ml of 4% OCT embedding medium (Electron Microscopy Sciences, Fort Washington, PA) and embedded to this compound in freezing chambers over 2-methyl butane (Sigma) by slowly freezing on liquid nitrogen. Cryostat cut frozen tissue sections were mounted on poly-L-lysine-coated slides (Fisher Scientific, Pittsburgh, PA) and stored at -70^0^C until stained. For performing IHC on paraffin-embedded tissues, sections were deparaffinized using a xylene gradient and rehydrated with an alcohol gradient ending in water. Endogenous peroxidase activity was blocked using 3% H_2_O_2_ and 0.1% NaN_3_ (Sigma) for 5 min. Nonspecific binding was prevented by preincubation of the slides with 10% goat serum (Biomeda Corporation, Foster City, CA) for 30 min. Proteinase K (Trevigen) was used for cell permeabilization before tissue staining. The specific Ab staining was performed according to the technical data sheets provided. Positive staining was visualized via three-step staining procedure with biotinylated rabbit anti-goat IgG (sc-2774) as the secondary Ab Streptavidin-HRP (Abcam Inc., Cambridge, MA) as the detection enzyme. All specific stainings were visualized using either DAB peroxidase substrate kit or NovaRed substrate for peroxidase (SK-4100 and SK-4800, both from Vector). Incubation steps for primary Abs were performed for 1 h whereas other steps lasted 30 min. At the end, the tissue slides were counterstained with hematoxylin.

For **Plasmacytoid Dendritic Cell Visualization**, anti-B220 and anti-GR1 Abs were used in addition to anti-CD11c and anti- I-Aβ^b^.
