# Supplementary material for: Expression of neuroimmune semaphorins 4A and 4D and their receptors in the lung is enhanced by allergen and vascular endothelial growth factor
Source: BMC Immunol. 2011 May 19;12:30. doi: 10.1186/1471-2172-12-30 (PMC3118960; doi:10.1186/1471-2172-12-30)
Supplement: Additional file 2 — Additional figure and figure legend. This section shows the photomicrographs of isotype control goat IgG stains of mouse lung tissues. [file 1471-2172-12-30-S2.DOC]

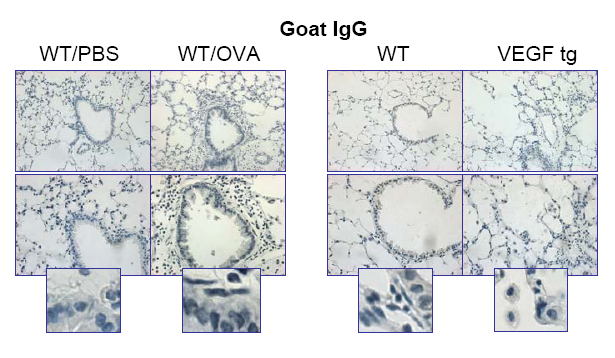
 **Figure legend**. Isotype control goat IgG stains of mouse lung tissues. (A) Formalin-fixed paraffin-embedded lung tissue sections obtained from PBS- and OVA-treated WT mice (left panel) were deparaffinized and immunohistochemistry was performed employing goat IgG as described in the Methods section . Right panel photomicrographs show immunohistochemistry on frozen lung tissue sections obtained from VEGF tg mice and control WT mice being on DOX water for 7 days.
